# Laparoscopic parenchyma-sparing resections for solid pseudopapillary tumors located in the head of pancreas

**DOI:** 10.1186/s12893-023-02028-0

**Published:** 2023-05-19

**Authors:** Zhengdong Zou, Lu Feng, Bing Peng, Jianhua Liu, Yunqiang Cai

**Affiliations:** 1 Divison of Pancreatic Surgery, Department of General Surgery, West China Hospital, Sichuan University, Chengdu, 610041 Sichuan China; 2 Department of Operation Room of Anesthesia Surgery Center, West China Hospital of Sichuan University, Chengdu, China; 3 Department of Hepatobiliary Surgery, The Second Affiliated Hospital of Hebei Medical University, Shijiazhuang, 050000 China

**Keywords:** Laparoscopy, Pancreatectomy, Enucleation, Duodenum-preserving pancreatic head resection

## Abstract

**Background:**

Solid pseudopapillary tumor (SPT) of the pancreas is a rare low-grade malignant tumor. Here, we aimed to determine the safety and feasibility of laparoscopic parenchyma-sparing pancreatectomy for SPT located in the pancreatic head.

**Methods:**

From July 2014 to February 2022, 62 patients with SPT located in the pancreatic head were operated laparoscopically in two institutions. These patients were divided into two groups according to the operative strategy: laparoscopic parenchyma-sparing pancreatectomy (27 patients, group 1) and laparoscopic pancreaticoduodenectomy (35 patients, group 2). The clinical data were retrospectively collected and analyzed in terms of demographic characteristics, perioperative variables, and long-term follow-up outcomes.

**Results:**

The demographic characteristics of the patients in the two groups were comparable. Compared to the patients in group 2, those in group 1 required less operative time (263.4 ± 37.2 min vs. 332.7 ± 55.6 min, p < 0.001) and experienced less blood loss (105.1 ± 36.5mL vs. 188.3 ± 150.7 mL, p < 0.001). None of the patients in group 1 had tumor recurrence or metastasis. However, 1 (2.5%) patient in group 2 showed liver metastasis.

**Conclusion:**

Laparoscopic parenchyma-sparing pancreatectomy is a safe and feasible approach for SPT located in the pancreatic head, with favorable long-term functional and oncological results.

Solid pseudopapillary tumor (SPT) of the pancreas is a rare neoplasm first reported by Frantz in 1959 [1]. It was formally named as SPT by WHO in 1996. SPT is a slow-growing and low-grade malignant tumor with a strong female predominance [2, 3]. Although it is a malignant tumor, the prognosis of SPT is favorable. Several studies have reported a 5-year survival rate of > 95% after tumor resection [4, 5].

Parenchyma-sparing resections of SPT located in the neck and body of the pancreas are safe and feasible [6, 7]. However, there is a paucity of data in the literature on the results of laparoscopic parenchyma-sparing resections of SPT located in the pancreatic head. In the present study, we included the largest number of laparoscopic parenchyma-sparing resections of SPT located in the pancreatic head. We aimed to determine the safety and feasibility of laparoscopic parenchyma-sparing resections of SPT by comparing this surgical approach with laparoscopic pancreaticoduodenectomy (LPD).

## Materials and methods

From July 2014 to February 2022, 62 patients with SPT located in the pancreatic head were operated laparoscopically in two institutions. Among them, 27 patients (group 1) underwent parenchyma-sparing pancreatectomy (including 21 patients with laparoscopic duodenum-preserving pancreatic head resection [LDPPHR] and 6 patients with enucleation), and 35 patients (group 2) underwent LPD. Before surgery, we fully inform each patient about the advantages and possible complications of two different surgical options. The final decision on the choice of surgical procedure is made jointly by the patient, their family members, and the surgeon. The clinical data were retrospectively collected by chart review and analyzed according to demographic characteristics (including age and sex), perioperative details (clinical symptoms, tumor size, tumor location, operative strategies, operative time, estimated blood loss, postoperative hospital stay, and complications), and follow-up outcomes (overall survival, tumor-free survival, and pancreatic endocrine/exocrine deficiency). The patients were followed up through interviews in the outpatient department and/or telephonic interviews. This study was approved by the Ethics Committee of the West China Hospital of Sichuan University (Approval No.: WCH 2018–97).

### Operative procedures

**Fig. 1:**
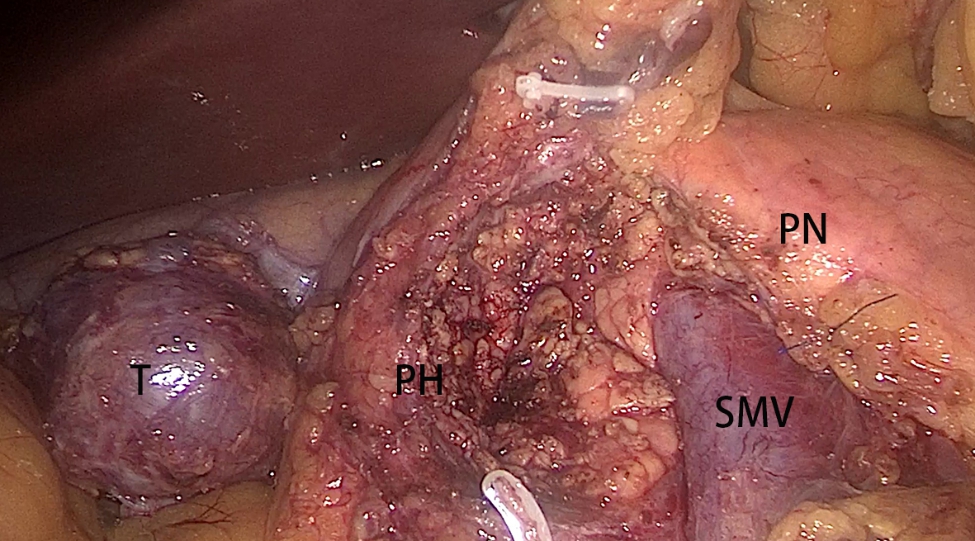
Laparoscopic enucleation for SPT. T: Tumor; PH: Pancreatic head; PN: Pancreatic neck; SMV: Superior mesenteric vein

**Fig. 2:**
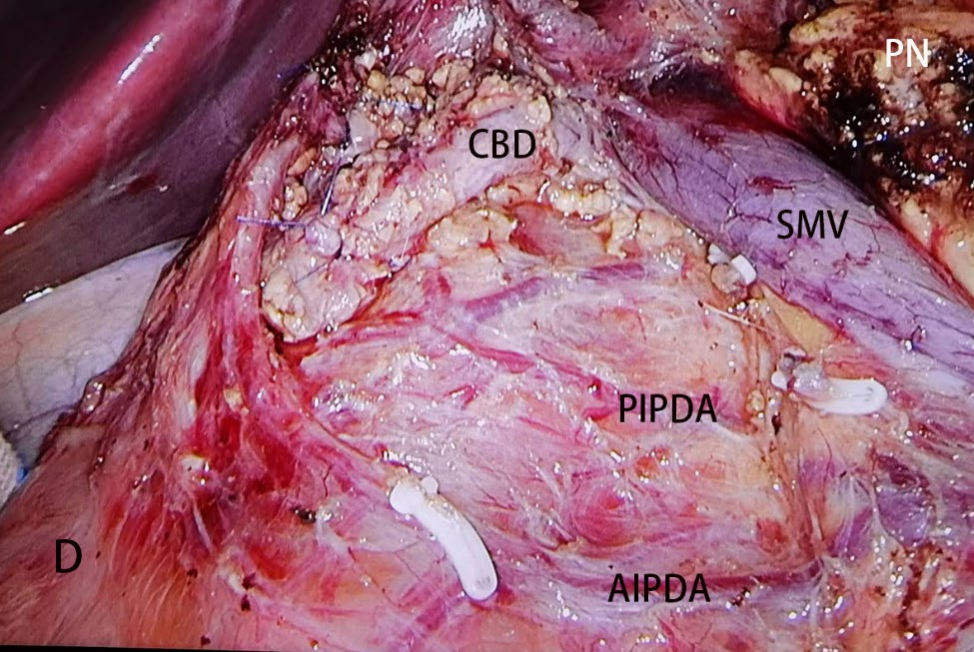
Laparoscopic duodenum-preserving pancreatic head resection for SPT. D: Duodenum; CBD: Common bile duct; PN: Pancreatic neck; SMV: Superior mesenteric vein. AIPDA: Anterior inferior pancreaticoduodenal artery; PIPDA: Posterior inferior pancreaticoduodenal artery

All patients received general anesthesia. The patients were placed in the supine position with their legs separated. Generally, five trocars were used. The observation trocar was located at the inferior umbilicus. Four trocars were placed symmetrically at the midclavicular line and the anterior axillary line. The surgery began with a careful examination of the entire abdominal cavity. For SPT located in the parenchyma of the pancreas, intraoperative ultrasonography was performed to identify the location and distance between the tumor and the main pancreatic duct. Enucleation was indicated for tumors located distant from the main pancreatic duct and the common bile duct (> 2 mm). Technically, enucleation involves tumor excision at the outer edge of the tumoral pseudocapsule to ensure complete resection (Fig. 1). Hemostasis was achieved by bipolar cautery or suturing. For tumors located very close to the main pancreatic duct and/or common bile duct that cannot be treated with enucleation, DPPHR was indicated. Briefly, the anterior inferior pancreaticoduodenal vessels, posterior inferior pancreaticoduodenal vessels, and posterior superior pancreaticoduodenal artery and its branches to the common bile duct were preserved. Almost all the pancreatic parenchyma in the pancreatic head was removed. Only a small part of the pancreatic parenchyma behind the common bile duct was preserved (Fig. 2). A Roux-en-Y, duct-to-mucosa pancreaticojejunostomy was performed. Drainage was routinely placed around the pancreatic anastomosis. Intraoperative frozen section examination of the resected sample was routinely performed to identify the tumor nature and confirm the negative resection margin. If the tumor was diagnosed as other pancreatic malignant tumors, the operative procedure was changed to LPD without any delay.

For LPD, the placement of trocars was the same as that described above. The pylorus-preserving PD with standard lymphadenectomy was adopted. The details of duct-to-mucosa pancreaticojejunostomy are described previously [8]. End-to-side hepaticojejunostomy was performed using 4–0/5–0 absorbable sutures. An end-to-side gastrojejunostomy/duodenojejunostomy was performed approximately 45 cm from the site of hepaticojejunostomy.

### Definitions

The 2016 update of the International Study Group [9] was followed to define the pancreatic fistula. Overall morbidity was defined as any complications associated with the operation within 90 days of surgery. Mortality was defined as death that was directly or indirectly associated with the operation within 90 days of surgery. The length of hospital stay was calculated from the day of surgery to the previous day of discharge.

### Statistical analysis

Statistical analyses were performed using SPSS 22.0 for Windows. Numerical data are expressed as mean ± standard deviation. Differences between variables were compared using the nonparametric Mann-Whitney *U* test, Student’s t-test, chi-square test, or Fisher’s exact test. A p-value of < 0.05 was considered to be statistically significant.

## Results

Table 1 shows the demographic characteristics of the patients included in this study. A total of 62 patients with SPT were enrolled, including 57 female patients (91.9%) and 5 male patients. The mean age of these patients was 34.7 ± 12.8 years. Thirty-eight patients (61.3%) were symptomatic, including 25 patients with abdominal pain and 13 patients with abdominal discomfort. Twenty-four patients (38.7%) were asymptomatic, and their pancreatic lesions were detected during routine examination using ultrasonography (US) or computed tomography (CT). No significant difference in age or sex proportion was observed between the two groups.

**Table 1:** The demographic characteristics and clinical symptoms

Variables	Group 1	Group 2	P value
No. of patients	27	35	-
Age (years)	33.6 ± 10.3	36.3 ± 13.7	0.37
Sex (F/M)	25/2	32/3	1.0
Clinical symptoms (n, %)			< 0.001
Abdominal pain	7 (25.9%)	18 (51.4%)	
Abdominal discomfort	7 (25.9%)	6 (17.1%)	
Without any symptoms	13 (48.2%)	11 (31.5%)	

Data are means with standard deviations in brackets or numbers with percentages in parentheses; F: female; M: male; NS: not significant

Table 2 shows the operative details of the patients. Compared to group 1 patients, group 2 patients required more operative time (263.4 ± 39.2 min vs. 332.7 ± 58.3 min, p < 0.001) and experienced higher blood loss (105.1 ± 36. mL vs. 388.3 ± 150.7 mL, p < 0.001). None of the patients in group 1 required blood transfusion, whereas 2 patients (5.7%) in group 2 required blood transfusion. One patient (2.9%) in group 2 required conversion of the laparoscopic approach to open surgery because of the involvement of the superior mesenteric vein. Group 2 patients had a larger tumor size than group 1 patients (4.6 ± 1.7 cm vs. 6.3 ± 2.7 cm, p = 0.09); however, the difference was not statistically significant.

**Table 2:** The operative outcomes

Variables	Group 1	Group 2	P value
Operative time (min)	263.4 ± 37.2	332.7 ± 55.6	< 0.001
Estimated blood loss (ml)	105.1 ± 36.5	388.3 ± 150.7	< 0.001
Pancreatic texture			1.0
soft	26	33	
hard	1	2	
Diameter of MPD (mm)	2.3 ± 0.3	2.7 ± 0.4	0.76
Conversion (n, %)	0	1, 2.9%	1.0
Tumor size (cm)	4.6 ± 1.7	6.3 ± 2.7	0.09

MPD: Main pancreatic duct

Table 3 shows the postoperative details. The mean postoperative hospital stay of group 1 patients was shorter than that of group 2 patients (9.1 ± 2.5 days vs. 15.1 ± 3.3 days, p = 0.025). The patients in both groups showed comparable time to oral intake (1.5 ± 0.7 days vs. 2.1 ± 0.8 days, p = 0.327). Ten patients (37.0%) in group 1 had postoperative complications, including 8 patients (29.6%) with clinically relevant pancreatic fistula (Grade B: 8 patients; Grade C: 0 patients), 1 patient with abdominal abscess, and 1 patient with abdominal bleeding. All complications, except one, were cured by conservative therapy. One patient with massive abdominal bleeding from the dorsal pancreatic artery on postoperative day 1 required re-operation. Twelve patients (34.3%) in group 2 had surgical complications, including 7 patients (20%) with pancreatic fistula (Grade B: 7 patients), 2 patients (5.7%) with delayed gastric emptying, 2 patients with abdominal abscess, and 1 patient with bile leakage. All the complications were cured by conservative therapy or US-guided percutaneous drainage. The overall complications of the patients in both groups were comparable. Pancreatic fistula occurred more in group 1 patients than in group 2 patients; however, the difference was not statistically significant (p = 0.38). There was no 90-day mortality in our series of patients.

**Table 3:** The post-operative details and follow-up outcomes

Variables	Group 1	Group 2	P value
Post-hospital stay (days)	9.1 ± 2.5	15.1 ± 3.3	0.025
Time to oral intake (days)	1.5 ± 0.7	2.1 ± 0.8	NS
Overall Complications (n, %)	11, 40.7%	12, 34.3%	0.60
Pancreatic fistula			0.73
Grade B	8 (29.6%)	7 (20%)	
Grade C	0	0	-
Abdominal abscess	1 (3.7%)	2 (5.7%)	1.0
Re-operation	1 (3.7%)	0	1.0
Abdominal bleeding	1 (3.7%)	0	1.0
DGE	0	2 (5.7%)	1.0
Bile leakage	0	1 (2.9%)	1.0
Tumor recurrence			1.0
Local recurrence	0	0	
Metastasis	0	1 (2.9%)	
Pancreatic function deficiency			
Exocrine	0	2 (5.7%)	1.0
Endocrine	0	1 (2.9%)	1.0

DGE: Delayed gastric emptying

All patients underwent US or CT every 6 months to 1 year after surgery. Follow-up data were collected by telephonic interviews or interviews in the outpatient department. The mean follow-up period was 46 months (range: 6–96 months). None of the patients in group 1 showed tumor recurrence or metastasis. The overall survival rate and tumor-free survival rate of group 1 patients were 100%. However, one patient (0.9%) in group 2 had liver metastasis and required radiofrequency ablation. During the follow-up period, none of the patients in group 1 developed endocrine or exocrine deficiency, whereas 2 patients (5.7%) and 1 patient (2.9%) in group 2 developed endocrine deficiency and exocrine deficiency, respectively.

## Discussion

SPT is a rare pancreatic entity that constitutes 1–3% of all pancreatic neoplasms [10]. It predominantly affects young female patients in their third or fourth decade of life [3, 11]. Thus far, < 10% of patients with SPT were males in the reported literature [12]. Although SPT is defined as a low-grade malignant pancreatic tumor, it has an excellent prognosis after curative resection.

Differing from its specific epidemiological characteristics, the clinical presentations of SPT are nonspecific. Abdominal pain or discomfort is the most common symptom, followed by gradual enlargement of the tumor mass and compression signs induced by the tumor mass. For tumors larger than 3 cm, the CT image of SPT is more specific, showing a well-circumscribed cystic and solid mass with heterogeneous enhancement, calcification of the mass, and no dilation of the main pancreatic duct or parenchymal atrophy. However, for tumors smaller than 3 cm, SPT may present as a solid mass. In the present study, most patients showed normal serum levels of carbohydrate antigen 19–9 and carcinoembryonic antigen. Only one patient in our series showed a slightly elevated level of carbohydrate antigen 19–9. Percutaneous or endoscopic fine needle aspiration may establish an accurate preoperative diagnosis [13]; however, this procedure may cause tumor dissemination and pancreatic inflammation [14]. Moreover, the efficacy of fine needle aspiration was also a debatable issue, and only 11 of the 24 (46%) patients who underwent fine needle aspiration were correctly diagnosed in Kim’s study [15]. Overall, the potential diagnosis of SPT may be established on the basis of its specific epidemiological characteristics and typical radiological presentations. However, a definite diagnosis should be established based on the results of pathological examination and immunohistochemical assays.

To date, the optimal treatment of SPT is complete resection [16]. Despite increased recognition of the characteristics of this tumor, the optimal operative strategy remains controversial. Typical pancreatectomy (PD, distal pancreatectomy) can be performed for treating SPT, resulting in a favorable oncological result. However, typical pancreatectomy is associated with the resection of a large portion of the normal pancreatic parenchyma, and this may result in exocrine and/or endocrine deficiency [17]. Furthermore, SPT mainly affects young patients with long life expectancy. Typical pancreatectomy is, however, associated with more detrimental effects.

Enucleation of the pancreatic tumor was first reported by Ernesto Tricomi in 1898. This procedure can maintain the normal anatomy of the upper digestive system and decrease the risk of postoperative exocrine and endocrine insufficiency [18]. It is widely applied to treat benign pancreatic tumors such as neuroendocrine tumors and cystic neoplasms [18–21]. However, laparoscopic enucleation for SPT has rarely been reported in the literature. To date, few retrospective studies with a small sample size have been conducted [22–24]. Consistent with previous studies, no patient who underwent enucleation in our series showed tumor recurrence and postoperative exocrine and endocrine insufficiency.

Enucleation is, however, not technically possible for all benign or borderline pancreatic tumors located in the pancreatic head [18]. If the distance between tumors and the main pancreatic duct is smaller than 2 mm, enucleation should be avoided [19]. DPPHR was first described by Beger in the 1980s [25]. Compared to PD, DPPHR can maintain the integrity of the duodenum and biliary tract, which is beneficial to the preservation of the endocrine and exocrine functions of the pancreas and helps prevent choledochojejunostomy-related complications [26]. It can also provide comparable long-term oncologic outcomes [27, 28].

Regarding the oncological results, none of the patients who underwent parenchyma-sparing pancreatectomy showed tumor recurrence, whereas one patient who underwent LPD developed liver metastasis. This result, however, does not imply that parenchyma-sparing pancreatectomy is associated with better oncological results. This difference may be caused by the fact that patients who underwent LPD had larger tumors, pancreatic parenchymal infiltration, and perineural or vascular infiltration.

The overall complications of patients who underwent laparoscopic parenchyma-sparing resection were higher than those in patients who underwent LPD, especially with regard to pancreatic fistula. The pancreatic fistula was more frequent in patients who underwent LDPPHR; this is mainly because LDPPHR is associated with excision of the pancreatic surface and anastomosis. Furthermore, the texture of the pancreas is soft, and the main pancreatic duct is not dilated, which are the risk factors of postoperative pancreatic fistula.

On the basis of our experience and previous study reports, several aspects are critical while performing parenchyma-sparing pancreatectomy for patients with SPT located in the pancreatic head. The lesion is considered to be SPT based on the preoperative evaluation. Tumors with suspected vascular involvement and peripancreatic organ involvement should be ruled out. For patients who are scheduled to undergo enucleation, the tumor must be at least 2 mm away from the main pancreatic duct based on preoperative radiological findings or intraoperative US examination; otherwise, LDPPHR is indicated. To ensure complete resection, enucleation should be performed through excision at the outer edge of the tumoral pseudocapsule. Intraoperative frozen section examination of the resected lesion and resection margins must be performed to identify the tumor nature and ensure that margins are tumor-free. If the frozen section examination reveals that the lesion belongs to other malignant tumors, conversion to a typical pancreatectomy is required without any delay.

There were several limitations associated with present study. It is a retrospective study with small sample size. We found that the parenchyma-sparing resections showed the advantage of lower exocrine and/or endocrine deficiency. However, these advantage was not statistically significant due to limited sample size. A prospective, randomized controlled trial (RCT) comparing parenchyma-sparing resections versus PD can provide valid pieces of evidence.

## Conclusions

SPT is a rare low-grade malignant tumor with a favorable prognosis. Although it is associated with higher overall complications, parenchyma-sparing pancreatectomy is safe and feasible for treating pancreatic SPT in well-selected patients, with favorable long-term functional and oncological results. More prospective multicenter studies with a larger sample size are required to establish a definite conclusion.

## Data Availability

All data generated or analyzed during this study are included in this published. article.

## Abbreviations

SPT: Solid pseudopapillary tumor
US: Ultrasonography
CT: Computed tomography
DPPHR: Duodenum-preserving pancreatic head resection
PD: Pancreaticoduodenectomy
LPD: Laparoscopic pancreaticoduodenectomy
LDPPHR: Laparoscopic duodenum-preserving pancreatic head resection
